# Comparative Study about Methods of Suicide between Japan and the United States

**DOI:** 10.2188/jea.14.187

**Published:** 2005-03-18

**Authors:** Toshiyuki Ojima, Yosikazu Nakamura, Roger Detels

**Affiliations:** 1 Department of Public Health, Jichi Medical School.; 2 Department of Epidemiology, School of Public Health, University of California, Los Angeles.

**Keywords:** Mortality, Suicide, Methods, Japan, United States

## Abstract

BACKGROUND: Suicide is one of the most important public health issues in both Japan and the United States. This study is to clarify the differences in methods of suicide between the two countries, among various races within the United States, and between genders and age-groups.

METHODS: Vital statistics mortality data and the estimated population in 1999 in Japan and in the United States were used. Age-adjusted mortality rates were calculated using the age-specific total population of Japan and the United States as a standard population. In addition, the proportionate distribution of suicide methods was calculated.

RESULTS: Age-adjusted mortality rates from suicide in Japan were about 2 times higher for males and 3 times higher for females compared with the United States. The most common method among both genders in Japan was hanging, followed by jumping from a high place. In the United States, it was firearms among both genders, followed by hanging among males and drugs among females. For Asians in the United States, hanging was the method of choice for about half among both genders; hanging was the most common method for the age group of 40 years or more among males and for all age groups among females. Firearms were the method of choice for the 20-39 age group among males.

CONCLUSIONS: Although the overall suicide rates among Asians in the United States were lower than Japan, the methods were similar to those in Japan.

Suicide is one of the most important public health issues in both Japan and the United States. Suicide causes about 30,000 deaths annually in the two countries, respectively. In 2000, it was the 6th cause of death in Japan and the 11th in the United States.^1^^,^^2^ Although suicide is often related to psychiatric disorders, it may be more preventable than other diseases and unintentional injuries. Therefore, suicide should be one of the major targets of public health efforts.

Psychological studies including psychological autopsy^3^ and suicide notes analyses^4^ have investigated the causes of suicide. Moreover, studies about the methods of suicide in relation to different cultural, ethnic, and age and gender groups can also provide useful information for developing effective intervention programs. Goldney^5^ reviewed suicide from the global perspective, and showed high rates in the Baltic States and countries of the Russian Federation, and low rates in some Mediterranean countries. Snyder^6^ compared methods of suicide used by Irish and Japanese, and found similarities despite cultural differences. However, very few studies have been conducted to compare suicide rates and methods between Japan and the United States, or to compare methods between all races in the United States.

The purpose of this study is to clarify the different methods of suicide between Japan and the United States, among various races in the United States, and to clarify differences between genders and age groups.

## METHODS

In the United States the International Classification of Diseases 10th Revision (ICD-10) was used first in 1999, whereas it was employed in 1995 in Japan. Therefore mortality data for 1999 were used for both Japan and the United States for this study.

For the data in Japan, the number of deaths from suicide by gender, age group, and ICD-10 code was obtained from vital statistics.^7^ For denominator data, The Current Population Estimates^8^ as of October 1, 1999, published by the Statistics Bureau, Ministry of Public Management, Home Affairs, Posts and Telecommunications, were used. Only the mortality data for Japanese who died within Japan and population data of Japanese who lived within Japan in 1999 were used for this study.

The “Multiple Cause of Death, 1999” published by the United States Department of Health and Human Services, National Center for Health Statistics (NCHS),^9^ was used for the United States. The cause-specific death numbers were collected by gender, age group, and race. Data for persons who died in the 50 states and the District of Columbia were used. Mortality data for foreign residents were excluded. For denominator data, the postcensal resident population in the United States in July 1, 1999, not including the Armed Forces overseas, was estimated based on the 1990 census.^10^

Age was categorized by five-year interval, although the oldest category was 85+ years. To identify the methods of suicide, the underling cause of death according to ICD-10 was used. The methods were recategorized into 11 groups: firearms (X72-74 for ICD-10), hanging (X70), drugs (X60-64), jumping from a high place (X80), gases (X66-67), drowning (X71), sharp objects (X78), pesticides (X68), smoke and fire (X76), jumping before a moving object (X81), and others (X65, 69, 75, 77, 79, 82-84). The rates were calculated individually by race for the United States. The race was categorized to five groups: Hispanics, non-Hispanic whites (Whites), African Americans (Blacks), Asians and Pacific Islanders (Asians), and American Indians and Alaska Natives (Natives).

Gender, age, race, and cause specific mortality rates were calculated. Age-adjusted mortality rates were calculated using the direct method, and the age-specific total population of Japan and the United States was used as a standard population. The percent distributions of methods of suicide were calculated based on the age-adjusted mortality rates.

## RESULTS

The total number of deaths from suicide was 31,413 (22,402 males and 9,011 females) in Japan and 29,180 (23,443 males and 5,737 females) in the United States. According to race in the United States, there were 24,634 suicide deaths among Whites (19,699 males and 4,935 females), 1,928 among Blacks (1,633 males and 295 females), 1,693 among Hispanics (1,428 males and 265 females), 647 among Asians (459 males and 188 females), and 278 among Native persons (224 males and 54 females).

**Figure 1.**
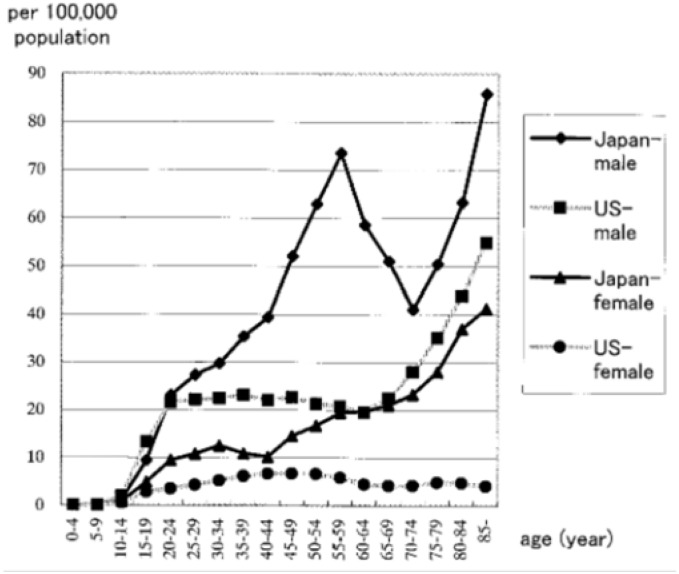
Suicide mortality rates by age-group and gender in Japan and the United States, 1999.

Suicide mortality rates by age group and gender in Japan and the United States are shown in Figure 1. The rates in Japan were higher than in the United States in most of the age groups for both genders. For males in Japan, there were two peaks, one for the 55-59-year age group the other for 85+ years. For females in Japan, the curve was almost monotonic, steadily increasing with age, except a very small peak in the 30-34-year age group. For males in the United States, the rates essentially plateaued in age groups of 20-69 years, and then rose sharply by age. For females in the United States, the curve was relatively flat, with a peak in the 40-54-year age groups.

Mortality rates by race, gender, and age group are shown in Table 1. Overall, age-adjusted mortality rates from suicide in Japan were almost twice as high for males and almost three times higher for females compared with the United States. The rates for males were about three times higher than females within Japan, and about four times higher within the United States. In the United States, descending rates were observed among Natives, Whites, Blacks, Hispanics, and Asians among males; and among Natives, Whites, Asians, Hispanics, and Blacks for females. Among non-White males, Hispanics and Asians had higher rates among elderly people; Blacks had a peak in the 20-39-year age groups; and Natives had high rates for ages 20-39 years. For non-White females, Asians had higher rates in the older age groups; Blacks and Natives had lower rates in the older age groups; and no suicide was observed among Native females aged 65 or more in 1999.

**Table 1.** Mortality rates by race, gender, and age-group. (1999, per 10,000 population)

Gender	Age (year)	Country and Race						

		Japan	United States					

			All	Whites	Blacks	Hispanics	Asians	Natives
Male	Total	34.1	18.7	20.9	11.1	10.9	10.5	22.2
	10-19	5.4	7.7	8.4	6.0	5.9	4.3	22.3
	20-39	29.1	22.3	25.0	18.4	13.7	14.1	41.9
	40-64	56.0	21.4	24.3	10.6	11.7	12.0	15.4
	65-	53.8	32.9	36.1	12.4	18.4	15.2	17.4

Female	Total	12.2	4.2	4.8	1.7	1.9	3.8	5.3
	10-19	2.9	1.7	1.8	1.1	1.4	1.9	4.2
	20-39	10.9	4.8	5.7	2.3	2.4	4.7	9.4
	40-64	15.6	6.2	7.3	2.3	2.4	4.0	6.5
	65-	27.4	4.4	4.8	1.5	2.2	6.9	-

Age was adjusted using the direct method from 5 year interval data. - : no case was observed

The percent distribution for methods of suicide by race and gender are shown in Table 2. The top two methods in Japan were hanging (70.4% for males and 60.0% for females) and jumping from a high place. Gases were the third among males, and drowning was the third among females. The most common method was firearms among both genders in the United States. The second most common method was hanging among males and drugs among females, and the third was drugs among males and hanging among females. When comparing males and females in the United States, the proportion for firearms was as high as two-thirds among males, while both firearms and drugs were about one-third among females. For each race in the United States, the three most common were the same among both genders for Whites, Blacks, Hispanics, and male Natives. For Asians, the most common method was hanging, at about half among both genders, and the second was firearms. The proportion jumping from a high place was also relatively high compared with other races. For female Natives, the three most common methods were drugs, hanging, and firearms.

**Table 2.** Proportions of methods of suicide by race and gender. (1999, %)

Gender	ICD-10 code and methods*		Country and Race						

			Japan	United States					

				All	Whites	Blacks	Hispanics	Asians	Natives
Male	X60-84	Total	100.0	100.0	100.0	100.0	100.0	100.0	100.0
	X72-74	Firearms	0.2	63.1	64.0	63.8	51.1	34.2	53.4
	X70	Hanging	70.4	18.2	17.0	18.5	32.7	41.5	35.1
	X60-64	Drugs	1.2	6.2	6.5	4.5	5.5	4.8	3.2
	X80	Jumping from a high place	8.3	2.1	1.9	3.5	2.5	7.2	0.0
	X66-67	Gases	6.6	5.2	5.8	1.9	1.9	1.4	2.8
	X71	Drowning	2.7	0.9	0.8	2.4	0.7	0.8	0.4
	X78	Sharp objects	2.6	1.4	1.3	1.3	1.6	3.5	2.0
	X68	Pesticides	2.4	0.1	0.0	0.1	0.0	0.6	0.0
	X76	Smoke and fire	2.4	0.5	0.4	0.7	1.0	2.2	0.5
	X81	Jumping before a moving object	2.3	0.8	0.7	1.6	1.3	2.0	0.4
	Others ^†^		1.1	1.5	1.5	1.7	1.7	1.9	2.4

Female	X60-84	Total	100.0	100.0	100.0	100.0	100.0	100.0	100.0
	X72-74	Firearms	0.0	37.2	38.5	36.1	30.5	13.7	25.6
	X70	Hanging	60.0	16.2	14.8	13.4	21.3	49.4	28.6
	X60-64	Drugs	2.8	29.2	30.2	21.8	27.6	13.1	34.4
	X80	Jumping from a high place	13.8	3.3	2.8	6.3	6.4	10.2	0.0
	X66-67	Gases	2.3	6.3	6.8	3.3	1.5	3.1	4.1
	X71	Drowning	7.6	1.9	1.5	6.0	2.7	4.4	2.4
	X78	Sharp objects	2.2	1.3	1.2	3.0	0.6	1.6	2.8
	X68	Pesticides	3.7	0.1	0.0	0.3	0.0	0.5	0.0
	X76	Smoke and fire	3.1	1.0	0.9	3.1	2.6	0.6	0.0
	X81	Jumping before a moving object	3.3	1.3	1.0	3.5	3.6	1.8	0.0
	Others ^†^		1.2	2.5	2.4	3.2	3.3	1.7	2.1

Proportion of age-adjusted mortality rates using direct method from 5 year interval data

* : Methods are provisional abbreviation. See ICD-10 code book for exact criteria of the methods.

† : X65,69,75,77,79,82-84 for ICD-10 code.

The proportions of selected methods of suicide by gender, age-group, and selected races are shown in Table 3. The proportion of hanging was the highest in the 65+ age group in Japan, in the 10-19 year age group in the United States, and in the 65+ age group among Asians. The proportion of firearms was the highest in the 65+ age group among males in the United States, and in the 10-19-year age group among Asian males.

**Table 3.** Proportions of selected methods of suicide by gender, age-group, and selected races. (%)

Country and race	ICD-10 code and methods*		Gender and age-group (year)							

			Male				Female			
	
			10-19	20-39	40-64	65+	10-19	20-39	40-64	65+
Japan	X72-74	Firearms	0.0	0.1	0.3	0.1	0.0	0.1	0.0	0.0
	X70	Hanging	72.8	63.0	70.3	78.4	55.8	49.1	58.7	70.8
	X60-64	Drugs	1.0	2.3	0.8	0.7	2.9	5.9	2.4	0.9
	X80	Jumping from a high place	16.9	14.3	6.4	5.2	26.7	26.5	11.8	4.7

United States	X72-74	Firearms	61.7	55.3	60.3	78.2	41.2	38.9	35.9	35.9
	X70	Hanging	29.2	25.6	15.9	9.0	34.2	18.7	11.0	20.2
	X60-64	Drugs	2.0	5.8	9.4	3.1	14.2	26.5	34.9	22.3
	X80	Jumping from a high place	1.8	2.5	2.0	1.8	3.8	3.2	2.8	4.6

Asians (in United States)	X72-74	Firearms	45.3	41.8	33.1	18.9	7.2	19.2	17.4	2.8
	X70	Hanging	45.3	33.3	42.6	54.1	42.9	42.7	39.7	72.9
	X60-64	Drugs	3.1	3.5	4.8	7.6	14.3	15.7	15.3	6.2
	X80	Jumping from a high place	3.2	8.4	5.2	9.3	21.5	2.3	10.3	18.1

Proportion of age-adjusted mortality rates using the direct method from 5 year interval data

* : Methods are provisional abbrevation. See ICD-10 code book for exact criteria of the methods.

## DISCUSSION

In general, the proportion of mortality from suicide is not very high among the elderly population, because mortality rates from other causes are high. The proportionate mortality due to suicide, however, was higher in older age-groups in Japan and among males in the United States, compared with younger age groups. Therefore, suicide is a major geriatric issue. Conwell, et al.^11^ found that the major independent risk factors for suicide in later life are depression and disruption of social structure. Public health measures to reduce these risk factors should be actively implemented for elderly people.

The suicide rates for males were much higher than for females in both Japan and the United States. Moller-Leimkuhler^12^ implied that traditional masculinity is a key risk factor for male vulnerability, and concluded that perceived reduction in social role opportunities leading to social exclusion could be the major reason for the gender gap.

The suicide rates among elderly were higher than among younger ages for both genders in Japan, but only for males in the United States. The rates among females in the United States did not differ by age. Though very few papers have focused on this point, several reasons can be considered. Tueth^13^ discussed the reasons of high suicide rate in elderly White males; he argued that older males have a less flexible and less diverse method of coping than females. Chaudron, et al.^14^ discussed antidepressant therapies and pointed out that females used outpatient health care and mental health care services more than males. Spicer, et al.^15^ focused on case fatality rates based on medical records; Kposowa^16^ calculated a relative risk of suicide for divorced males and females; their results, however, could not completely explain this figure. Further researches are required to this point.

Japan had higher suicide rates than the United States. Araki, et al.^17^ implied that high suicide rates among middle-aged men resulted from economic decline. Boor^18^ found a positive temporal association between unemployment and suicide in six of eight countries. Several studies focused on cultural issues. Iga et al.^19^^,^^20^ discussed various cultural issues, including “means-end” discrepancy; that is, a person who failed to succeed in spite of his efforts, including unquestioning loyalty, predicted suicide. Domino, et al.^21^ observed that Japanese, compared with Americans, tended to think that an individual has the right to die, and that suicide is a normal behavior, according to a survey of Japanese and American medical students. Though the higher suicide rates could be reflected by the higher prevalence of depression, Kessler et al.^22^ showed lower prevalence of mood disorder (3.1%) as well as substance disorder (1.7%) in Japan than in the United States (9.6% and 3.8%, respectively). The other possible factor is religion. American people may be more religious than Japanese; they would less consider committing suicide; or they would reform their mind through religious activities even if they once think about suicide. Nisbet, et al.^23^ observed 4.34 of odds ratio who never participate in religious activities among suicide victims compared with natural death. Stack^24^ observed that 1.94% and 5.62% of published books are religious books in Japan and the United States respectively; however, he failed to find clear relationship between religiosity, measured by percentage of religious books, and suicide rates after controlling for other factors in the study, which included 25 nations. Collaborative studies incorporating sociology and epidemiology are needed to clarify and quantify the cultural factors responsible for the higher suicide rates in Japan.

Suicide rates from individuals using firearms were higher in the United States than in Japan. Firearms are commonly available in the United States, while only a few restricted people in Japan are allowed to possess firearms. Romero et al.^25^ reported that the rates of firearm suicide have consistently exceeded rates of firearm homicide during the past two decades. Wintemute et al.^26^ conducted a cohort study and concluded that the purchase of a handgun is a substantial risk factor for suicide. An important question is whether restriction of the tools for suicide can reduce suicide. Gunnell, et al.^27^ observed a decrease in suicide rates after detoxification of domestic gas, which is reduction in its carbon monoxide and lethality. Lester, et al.^28^ reported that requiring prescriptions for sedatives and hypnotics in Japan led to a decrease in their use for suicide, but did not lead to an increase in the use of other methods. A higher suicide rate was observed among 10-19 years-old males in the United States than Japan in our study; one of the causes of this observation might be the common availability of firearms in the United States. Overall suicide rates, however, were higher in Japan than in the United States despite tight restrictions of firearms use. Although restriction of available methods has some effect on suicide rates, other factors seems to have important effects on overall suicide rates.

When comparing Asians in the United States with those in Japan, the overall suicide rates were much lower. Methods of suicide, however, had some similarities: high proportions of hanging and jumping from a high place compared with other races in the United States. He, et al.^29^ reported that the leading method of suicide was hanging in some regions, while it was poison in other regions of China. Although the overall suicide rates are influenced by rapidly changeable factors, selection of methods might be affected by stable factors, e.g. cultural background.

When comparing age groups of Asians within the United States, firearms were common amongst younger males, while hanging was common in older age groups. There may be some birth cohort effects on this difference. Although the first generation of immigrants is highly influenced by the culture of their native countries, the second or later generation is thought to be more strongly affected by the culture in the United States.

Some study limitations should be kept in mind. Vital statistics mortality data based on death certificates were used in this study in both Japan and the United States. Death certificates, which were issued within limited information, might occasionally be different from the truth. Another limitation is the possibility of misclassification of race in vital statistics data and an undercount for some minority populations in the United States, as Rosenberg, et al.^30^ pointed out. Finally, only the data of Japan and the United States were analyzed in this study, and the data of other Asian countries such as China or Korea, or other countries, which are the origins of many American people, were not compared.

In summary, elderly people had higher suicide rates among both genders in Japan, and among males in the United States; however, suicide rates were almost same between elderly and younger people among females in the United States. Japan had higher suicide rates than the United States. The most common method was hanging in Japan, and firearms in the United States. Although the overall suicide rates among Asians in the United States were lower than in Japan, the methods tended to be similar.
